# Utilizing the Transverse Thermoelectric Effect of Thin Films for Pulse Laser Detection

**DOI:** 10.3390/s22134867

**Published:** 2022-06-28

**Authors:** Yanju Sun, Haorong Wu, Lan Yu, Hui Sun, Peng Zhang, Xiaowei Zhang, Bo Dai, Yong Wang

**Affiliations:** 1 School of Space Science and Physics, Shandong University, Weihai 264209, China; 201900830051@mail.sdu.edu.cn (Y.S.); huisun@sdu.edu.cn (H.S.); zhangpeng@sdu.edu.cn (P.Z.); 2 Faculty of Materials Science and Engineering, Kunming University of Science and Technology, Kunming 650093, China; wuhaorong@stu.kust.edu.cn (H.W.); 11302084@kust.edu.cn (L.Y.); 3 State Key Laboratory for Environmental-Friendly Energy Materials, Southwest University of Science and Technology, Mianyang 621010, China; xiaoweizhang@swust.edu.cn (X.Z.); daibo@swust.edu.cn (B.D.)

**Keywords:** transverse thermoelectric effect, anisotropic seebeck coefficients, pulse laser detectors, thin films, fast response

## Abstract

In this work, pulse laser detectors based on the transverse thermoelectric effect of YBa_2_Cu_3_O_7-δ_ thin films on vicinal cut LaAlO_3_ (001) substrates have been fabricated. The anisotropic Seebeck coefficients between *ab*-plane (*S_ab_*) and *c*-axis (*S_c_*) of thin films are utilized to generate the output voltage signal in such kind of detectors. Fast response has been determined in these sensors, including both the rise time and the decay time. Under the irradiation of pulse laser with the pulse duration of 5–7 ns, the output voltage of these detectors shows the rise time and the decay time of 6 and 42 ns, respectively, which are much smaller than those from other materials. The small rise time in YBa_2_Cu_3_O_7-δ_-based detectors may be due to its low resistivity. While the high thermal conductivity and the large contribution of electronic thermal conductivity to the thermal conductivity of YBa_2_Cu_3_O_7-δ_ are thought to be responsible for the small decay time. In addition, these detectors show good response under the irradiation of pulse lasers with a repetition rate of 4 kHz, including the precise determinations of amplitude and time. These results may pave a simple and convenient approach to manufacture the pulse laser detectors with a fast response.

## 1. Introduction

Laser-irradiation sensors are of great value in detecting the laser parameters, such as power/energy, pulse duration, pulse frequency and shape. These measurements may be required as closed loop control arrangement or simple record-keeping. Nowadays, the commonly used laser-irradiation sensors are represented by three types according to their physical principles, such as photodiodes, pyroelectric and thermoelectric sensors [1].

The photodiode-based sensors detect lasers by converting photon energy into electron–hole pairs in p-n junctions, giving rise to the fast response time of sub-nanoseconds. In addition, the sensitivity of this type of sensors is much higher, which allows the detectability of low power continuous wave (CW) and pulse lasers [1,2,3]. However, such a high sensitivity is usually companied with a relatively low power saturation threshold, which is not suitable for the detection of high power lasers. In addition, these photodiode-based sensors suffer from the limited spectral response, which is determined by the band gap of the semiconductors. For instance, sensors based on silicon possess a spectral response range between 0.2 and 2 µm [4].

Pyroelectric sensors transduce a temperature change into an electric signal via the spontaneous polarization. Benefiting from their thermal nature, pyroelectric sensors have the broad spectral response (i.e., from UV to THz). High sensitivity for pulse lasers can also be achieved in such pyroelectric sensors. Unfortunately, due to the transient response on the temperature, this type of sensors allows just measurements of pulse lasers with the response time of microseconds [5,6].

Thermoelectric sensors are subdivided into two groups, including the devices utilized the traditional longitudinal thermoelectric (LTE) effect and the ones based on the transverse thermoelectric (TTE) effect [7,8,9]. The common advantages of these thermoelectric sensors include the wide spectral response range and the high saturation threshold of laser irradiation. The standard LTE sensors are composed of electrically connected thermocouples, where the electrical and thermal flows are parallel. Due to the existence of thermal resistance layer and the size of thermoelectric legs, this type of LTE sensor gives the response time of seconds [10].

**Figure 1:**
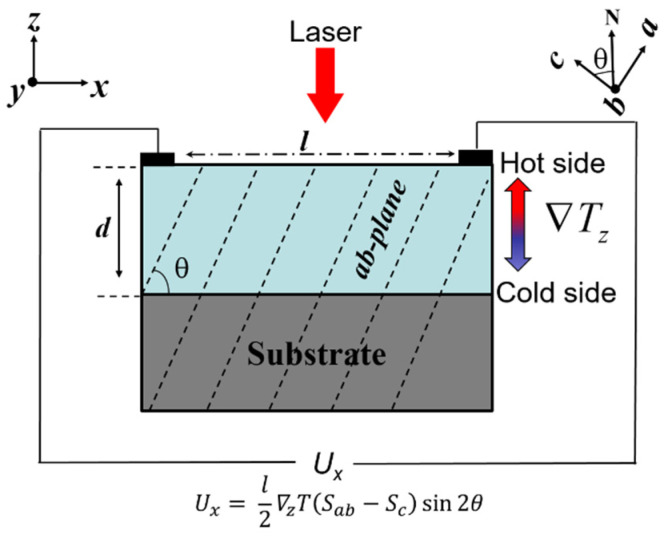
Schematic structure of laser detectors based on the TTE effect of thin films (in blue) grown on vicinal cut substrate (in black).

TTE sensors utilize the anisotropic Seebeck coefficients, which exhibits unique properties compared to the LTE effect. By way of example, one sole material with significant anisotropy could produce a TTE voltage [8,9,11], where at least two different materials are required to fabricate the thermocouples in LTE sensors [10]. This means much more straightforward device fabrication is available in TTE sensors with a much lower cost. In addition, the electrical and thermal flows are perpendicular to each other in TTE effect, which indicates the relatively independent management of heat and electric fluxes. For instance, an ultrafast response time of nanoseconds (ns) can be achieved by sacrificing the thickness of sensors, while the voltage signal magnitude can be maintained by elongating the length [9]. A general approach to fabricate TTE sensors is growing epitaxial thin films with anisotropic Seebeck coefficients on vicinal cut substrates to engineer a tilted angle (*θ*) between *c*-axis orientation and surface normal of thin films. When a laser irradiates on the top surface of thin film, a temperature gradient along the out-of-plane direction (on the *z*-axis) of thin film will be generated, and then produce a voltage in the in-plane direction (on the *x*-axis) due to different Seebeck coefficients between *ab*-plane (*S_ab_*) and *c*-axis (*S_c_*) of thin film (see Figure 1). The output in-plane voltage (*U_x_*) induced by the TTE effect in the film surface is expressed in the following form:

(1) $$\;\;U_{x}=\frac{l}{2}\sin\left(2\theta \right)\left(S_{ab}-S_{c}\right)\nabla T_{z}$$

where $\nabla T_{z}$ is the temperature gradient along film thickness direction, *l* is the irradiation length, and *d* is thickness of thin film [8,12,13,14,15,16]. However, most of the previous studies on the TTE-based laser detectors just focused on the enhancement of voltage sensitivity, and less attention has been paid to the response speed. In this work, we investigate the fast response of pulse laser detectors based on the TTE effect of YBa_2_Cu_3_O_7-δ_ (YBCO) thin films grown on vicinal cut LaAlO_3_ (001) substrates.

## 2. Materials and Methods

YBCO thin films (10 × 5 mm) with thickness of about 200 nm were used as the sensing layers, which were deposited on 15° vicinal cut LaAlO_3_ (LAO) (001) single crystal substrates by pulse laser deposition (PLD). The PLD system included a KrF excimer pulse laser with a wavelength of 248 nm and pulse duration of 20 ns. Laser fluence and pulse frequency were fixed at ~1.8 J cm^−2^ and 3 Hz, respectively. A polycrystalline YBCO target was used for the ablation process. During the deposition process, the substrate temperature and flowing oxygen pressure were set at around 785–800 °C and 40–45 Pa, respectively. Then, an oxygen annealing was performed at 500 °C with the oxygen pressure of 100,000 Pa for 1 h. The phase structure of thin film was checked by an X-ray diffraction (XRD, Rigaku D/MAX 2500V/PC X). The microstructure of YBCO thin film on vicinal cut substrate was studied by a transmission electron microscopy (TEM, JEOL ARM 200F). The schematic structure of laser detectors based on the TTE effect of thin films is shown in Figure 1. Two Pt electrodes were deposited on the top surface of YBCO thin films with the irradiation length of *l* = 7 mm along the inclined direction. Two types of pulse lasers have been used as the irradiation sources to measure the TTE voltage response. An optical parametric oscillator (OPOTEK INC) was employed to generate pulse laser with the repetition rate of 1 Hz, the wavelength of 1000 nm, and the pulse duration of about 5–7 ns, while a solid state Q-switched laser at 1064 nm (MPL-H-1064) was utilized to produce the pulse laser with the repetition rate of 4 kHz and the pulse duration of ~10 ns. The voltage signals of these TTE-based laser detectors were recorded by an oscilloscope (Rohde & Schwarz RTE 1102, 1 GHz bandwidth).

## 3. Results

**Figure 2:**
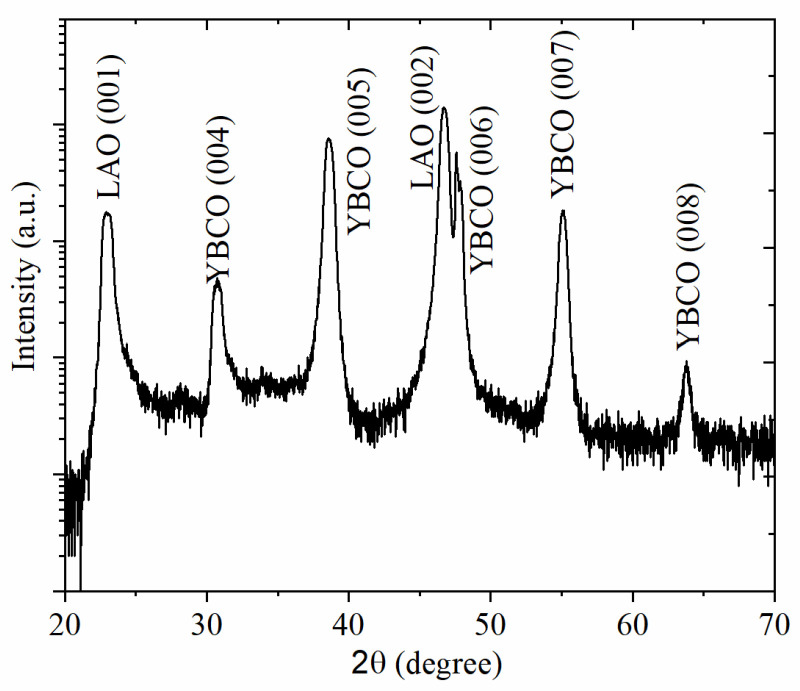
XRD pattern YBCO thin film grown on 15° vicinal cut LAO (001) substrate.

The XRD pattern of θ–2θ scan of YBCO thin film grown on 15° vicinal cut LAO (001) substrate is shown in Figure 2. Besides the diffraction peaks of LAO (001) substrate, only (00*l*) diffraction peaks of YBCO thin film are detected, while other orientations are not observed. This demonstrates the pure phase and the *c*-axis tilted growth of YBCO thin films.

**Figure 3:**
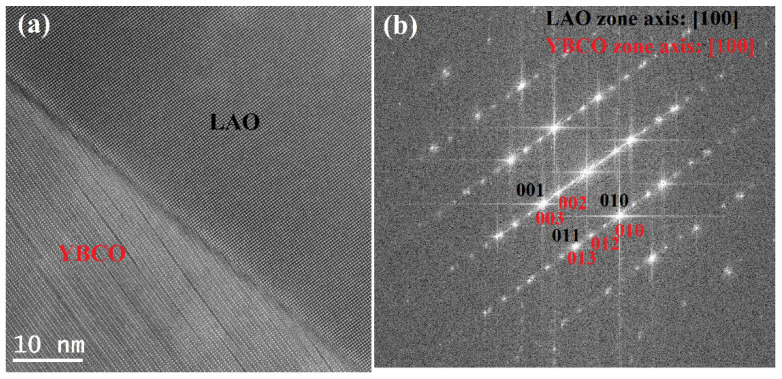
(**a**) HRTEM image of YBCO thin film on 15° vicinal cut LAO and (**b**) the corresponding FFT pattern.

The microstructure of tilted YBCO thin film has been studied by TEM. Figure 3a shows the high-resolution TEM (HRTEM) image of YBCO thin film on 15° vicinal cut LAO (001). It is clearly seen in Figure 3a that YBCO thin film exhibits the typical layered characteristics, which is well consistent with its crystal features with the alternative stacks of CuO_2_, BaO, CuO and Y layers along the *c*-axis [17]. Such layered structures give the large Seebeck coefficient anisotropy of (*S_ab_* − *S_c_*) = 30 µV/K [18], which is responsible for the origin of TTE voltage. In addition, the interface between YBCO thin film and LAO substrate is sharp, indicating the high quality of YBCO thin film. The fast Fourier transform (FFT) pattern of Figure 3a is presented in Figure 3b. The FFT pattern of YBCO thin film shows the diffraction features of single crystal, which confirms the high crystal quality of YBCO thin film again. Considering the crystal constants of LAO (*a* = 0.3821 nm) and YBCO (*a* = 0.3821, *b* = 0.3887 and *c* = 1.169 nm), the FFT patterns of YBCO and LAO are well indexed in red and black, respectively (see Figure 3b). These results demonstrate that single crystal YBCO thin film is epitaxial growth on 15° vicinal cut LAO substrate, with the crystallographic orientation relationships [100]_YBCO_ ǁ [100]_LAO_ (in-plane)_,_ [010]_YBCO_ ǁ [010]_LAO_ (in-plane) and [001]_YBCO_ ǁ [001]_LAO_ (out-of-plane).

**Figure 4:**
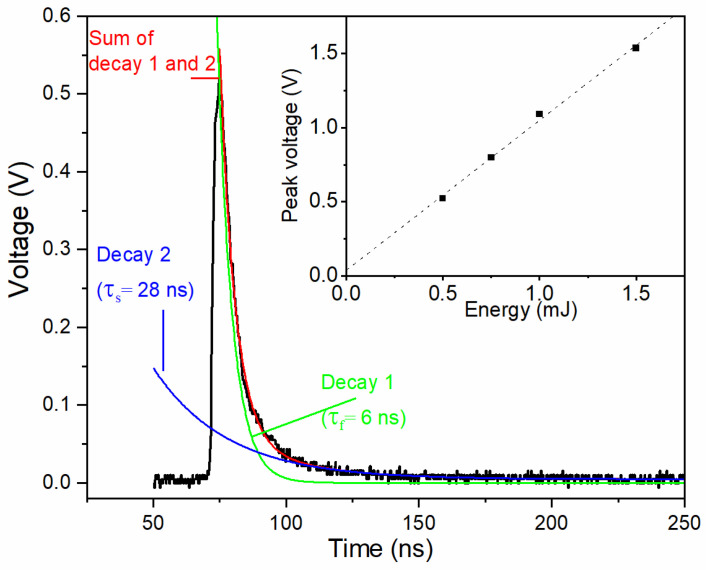
TTE voltage signal of YBCO thin film laser detector under the irradiation of pulse laser with the wavelength of 1000 nm and the repetition rate of 1 Hz. Exponential decay fits for decay time are shown, including the fast component of decay 1 (in green), the slow component of decay 2 (in blue), and the sum of decay 1 and 2 (in red). The inset shows the linear relationship between peak voltage and pulse energy.

The voltage response of YBCO thin film laser detector under the irradiation of pulse laser with the repetition rate of 1 Hz, the wavelength of 1000 nm, the pulse duration (*τ*_p_) of 5–7 ns, and the energy of 0.5 mJ is shown in Figure 4. As seen in Figure 4, the TTE voltage of YBCO thin film exhibits the rise time (*τ*_r_, 0–100% of peak value) of about 6 ns, which is much faster than other reported ones, such as 7 ns in La_0.5_Sr_0.5_CoO_3_ thin film, 33–100 ns in Ca_3_Co_4_O_9_ thin film [19], 51 ns in La_0.9_Ca_0.1_MnO_3_ thin film [20], and 33 ns in CuCr_0.98_Mg_0.02_O_2_ thin film [21]. Such a fast response speed is related with the low room-temperature resistivity of YBCO thin film (around 5.6 × 10^−4^ Ohm cm). It is believed that the low resistivity yields small optical penetration depth, and the rise time has a monotonous increasing relationship with this penetration depth [9]. In addition, the inset of Figure 4 indicates a linear relationship between peak voltage and pulse energy.

In addition, the decay time (*τ*_d_, 100–0% of peak value) in Figure 4 is about 42 ns, which is significantly smaller than the ones of 1000 ns in Ca_3_Co_4_O_9_ thin film [19], 4000 ns in La_0.9_Ca_0.1_MnO_3_ thin film [20], and more than 7000 ns in CuCr_0.98_Mg_0.02_O_2_ thin film [21]. The *τ*_d_ means a recovery process of the TTE sensors from a large temperature difference to a normal state without temperature difference due to the thermal diffusion. Early studies just deemed that the *τ*_d_ is inversely proportional to the total thermal conductivity (*k*_total_), with the expression of τ_d_ ∝ *d*^2^*c*/2*k*_total_ (where *c* is the density of thin film) [22]. More recently, a detailed analysis showed that the composition of *k*_total_ is also of great value to tune the *τ*_d_, in addition to the *k*_total_ [21]. Generally, *k*_total_ includes electronic thermal conductivity (*k*_e_) and lattice thermal conductivity (*k*_l_), written as *k*_total_ = *k*_e_ + *k*_l_. Meanwhile, *k*_e_ and *k*_l_ may dominate the corresponding fast (*τ*_f_) and slow (*τ*_s_) components of the decay signal, respectively. Once the *k*_e_/*k*_total_ is small, the decay signal may exhibit a long voltage tail down to zero, which increases the total *τ*_d_ significantly. Hence, both *k*_total_ and *k*_e_/*k*_total_ have notable influences on *τ*_d_. The smaller *τ*_d_ of 42 ns in YBCO, including the fast component *τ*_f_ of 6 ns (decay 1 in Figure 4) and the slow part *τ*_s_ of 28 ns (decay 2 in Figure 4), may originate from its room temperature *k*_total_ of 6 W/(m·K) and *k*_e_/*k*_total_ of 33.3% [23]. In the case of CuCr_0.98_Mg_0.02_O_2_ thin film, its room temperature *k*_total_ is as large as 8.8 W/(m·K), but its *τ*_d_ remains larger than 7000 ns surprisingly [21]. The fitting on the decay time gives the fast component of 30 ns and the slow part of about 5000 ns, which may be related with its low *k*_e_/*k*_total_ of 0.02% [21]. With respect to Ca_3_Co_4_O_9_, *k*_total_ is just 2.4 W/(m·K), but the *k*_e_/*k*_total_ is as high as 58.3% [24]. Such a large *k*_e_/*k*_total_ may be responsible for its medium *τ*_d_ of 1000 ns with the fast component of 124 ns and the slow part of 583 ns. Similar situations also exist in La_0.9_Ca_0.1_MnO_3_ thin film with the *τ*_d_ of 3500 ns, *τ*_f_ of 276 ns and the *τ*_s_ of about 1400 ns, which may be from its *k*_total_ of 1 W/(m·K) and *k*_e_/*k*_total_ of 80% [25]. The summarized details of the decay features of TTE voltage signals (including *τ*_d_, *τ*_f_, and *τ*_s_), the thermal conductivity (*k*_total_), and the contribution of electron thermal conductivity (*k*_e_/*k*_total_) of these materials are shown in Table 1. 

Here, it is worth noting that both *τ*_r_ and *τ*_d_ of laser detectors should be considered for the detection of pulse lasers with repetition frequencies larger than 1 Hz, as *τ*_d_ will strongly affect the measurements of the following pulses. However, most of the previous works only focus on the *τ*_r_ and neglect the influences of *τ*_d_ on the maximum measurable frequency of a train of pulse lasers. According to the discussion above, it is seen that the combinational properties of thin films, including the low resistivity, the high thermal conductivity and the large contribution of electronic thermal conductivity to the thermal conductivity, are beneficial to a fast and broadband response. Among the various materials, YBCO exhibits better properties comprehensively than other materials, giving rise to much faster response with smaller *τ*_r_ and *τ*_d_ simultaneously.

**Figure 5:**
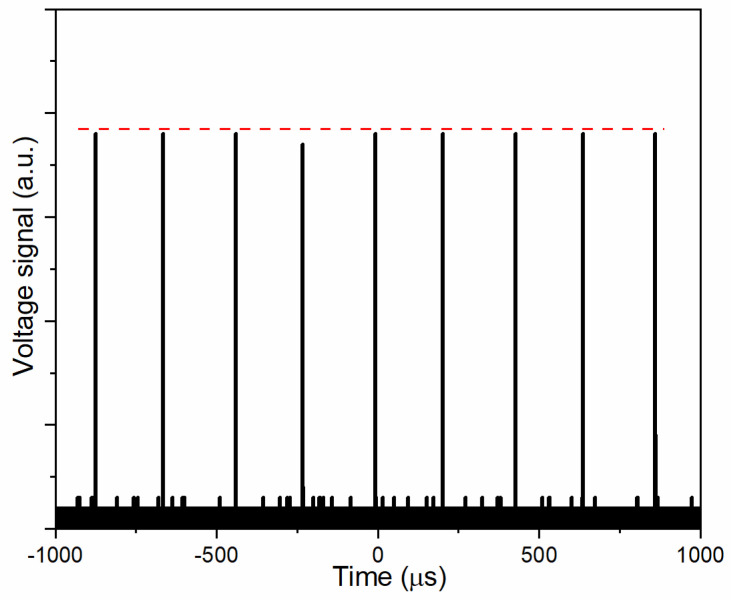
The voltage response as a function of time when the YBCO thin film laser detector is irradiated by laser pulses with a repetition rate of 4 kHz. The dashed line in red indicates the variation of amplitudes of different signals.

The response of YBCO thin film laser detector under the irradiation of a train of pulse lasers (*τ*_p_~10 ns) with a repetition rate of 4 kHz is shown in Figure 5. As seen in Figure 5, pulse signals with the interval of about 230 µs are clearly observed, which is quite close to the theoretical value of 250 µs. In addition, the amplitude of these signals shows the high precision with the standard deviation of 0.0734. These results demonstrate that the YBCO-based TTE sensors have the ability to measure the pulse lasers with a high repetition rate.

## 4. Conclusions

In summary, we have fabricated the pulse laser detectors based on the transverse thermoelectric effect of YBa_2_Cu_3_O_7-δ_ thin films on miscut LaAlO_3_ (001) substrates. The Seebeck coefficient anisotropy between *ab*-plane (*S_ab_*) and *c*-axis (*S_c_*) of YBa_2_Cu_3_O_7-δ_ thin films is utilized to generate the output voltage via the transverse thermoelectric effect, in contrast to the traditional longitudinal thermoelectric effect. These transverse thermoelectric detectors exhibit the fast response, including both the rise time and the decay time. Irradiated by pulse laser with the pulse duration of 5–7 ns, the rise time and the decay time of the output voltage are 6 and 42 ns, respectively. Compared with other materials, these values of YBa_2_Cu_3_O_7-δ_ thin film are significantly smaller. Such a fast rise time may come from the low resistivity of YBa_2_Cu_3_O_7-δ_ thin films, while the small decay time may originate from its high thermal conductivity and the large contribution of electronic thermal conductivity to the thermal conductivity. In addition, these detectors are able to precisely measure the pulse lasers with a repetition rate of 4 kHz. Since this kind of laser detector based on the transverse thermoelectric effect of thin film possesses the characteristics of fast response and wide bandwidth, we believe that this work may provide a roadmap to fabricate the thin film laser detectors with high performances. 

## Figures and Tables

**Table 1:** The summarized details of signal decay time and thermal conductivity of various materials.

Materials	*τ*_d_ (ns)	*τ*_f_ (ns)	*τ*_s_ (ns)	*k*_total_ (W/(m·K))	*k*_e_/*k*_total_ (%)	Reference
YBa_2_Cu_3_O_7-δ_	42	6	28	6	33.3	This work and [23]
Ca_3_Co_4_O_9_	1000	124	583	2.4	58.3	[19,24]
La_0.9_Ca_0.1_MnO_3_	3500	276	1400	1	20	[20,25]
CuCr_0.98_Mg_0.02_O_2_	7000	30	5000	8.8	0.02	[21]

## Data Availability

MDPI Research Data Policies.
